# Tunable Spun Fiber Constructs in Biomedicine: Influence of Processing Parameters in the Fibers’ Architecture

**DOI:** 10.3390/pharmaceutics14010164

**Published:** 2022-01-11

**Authors:** Catarina S. Miranda, Ana Francisca G. Silva, Sílvia M. M. A. Pereira-Lima, Susana P. G. Costa, Natália C. Homem, Helena P. Felgueiras

**Affiliations:** 1 Centre for Textile Science and Technology (2C2T), Campus of Azurém, University of Minho, 4800-058 Guimarães, Portugal; catarina.miranda@2c2t.uminho.pt; 2 Center of Chemistry (CQ), Campus of Gualtar, University of Minho, 4710-057 Braga, Portugal; pg40181@alunos.uminho.pt (A.F.G.S.); silviap@quimica.uminho.pt (S.M.M.A.P.-L.); spc@quimica.uminho.pt (S.P.G.C.); 3 Digital Transformation CoLab (DTx), Building 1, Campus of Azurém, University of Minho, 4800-058 Guimarães, Portugal; natalia.homem@dtx-colab.pt

**Keywords:** drug delivery, electrospinning, fiber architectures, fibrous constructs, tissue engineering, wet-spinning

## Abstract

Electrospinning and wet-spinning have been recognized as two of the most efficient and promising techniques for producing polymeric fibrous constructs for a wide range of applications, including optics, electronics, food industry and biomedical applications. They have gained considerable attention in the past few decades because of their unique features and tunable architectures that can mimic desirable biological features, responding more effectively to local demands. In this review, various fiber architectures and configurations, varying from monolayer and core-shell fibers to tri-axial, porous, multilayer, side-by-side and helical fibers, are discussed, highlighting the influence of processing parameters in the final constructs. Additionally, the envisaged biomedical purposes for the examined fiber architectures, mainly focused on drug delivery and tissue engineering applications, are explored at great length.

## 1. Introduction

In recent years, micro- and nanofibers have emerged as promising tools for biomedical applications for displaying advantageous features, including large surface area, high porosity, and tunable structure, functionality, mechanical performance [1]. Moreover, their high area-volume ratio (relation between width and diameter), capacity to form intricated 3D-networks, and ability to incorporate various chemical functions or tune their molecular orientation to improve bioactivity, make them ideal for drug delivery and tissue engineering uses [1,2]. Polymer physical and chemical properties can be improved during fiber extrusion by ameliorating the alignment of the polymeric chains through the fiber axis. This effect can be achieved via spinning techniques, which are specialized extrusion methods in which a spinneret forms continuous filaments [3]. These techniques involve irreversible processes, in which the solidification of a liquid with a restricted size occurs in two directions. Usually, the goal is to convert the solid polymer in a spinnable solution, either by melting or dissolution in appropriate solvent or by chemically altering the polymer to generate soluble derivatives [4]. The most commonly used spinning methods are the electrospinning, wet-spinning, dry-spinning and melt-spinning [5].

In the melt-spinning technique, dried polymer granules are melted inside an extruder and used as the spinning dope. Afterwards, the filament is subjected to a fast fiber solidifying process due to a one-way heat transfer [6]. This manufacturing process is preferred amongst many polymers, since no solvents are required [7]. However, there are still several limitations associated with this technique, such as polymer decomposition at temperatures below the melting point, a weak control of the temperature of the polymer melt, and restrictions in the ability to produce fine fibers [8]. Electrospinning resorts to electrostatic forces to induce the formation of fine fibers from polymer solutions [9]. In this process, the polymer solution is ejected through a needle connected to an electric field, which attracts the polymer jet towards a collector [10]. Electrospinning has been gaining considerable attention in biomedicine, due to the versality, simplicity and cost efficiency of the technique. Further, the resemblance of the electrospun fibers with the organization of the extracellular matrix can contribute to higher proliferation and migration of cells, as well as an improved control of fluid loss [11,12]. Electrospun fibers can also present high loading and encapsulation efficiency, making them suitable for drug release systems [13]. Still, there are as well some issues associated with the use of these electrospun constructs, mainly the limited cell infiltration throughout the innermost regions of the scaffolds that cannot be ensured via these high fiber packing density and small pore-size constructs and the maintenance of environments that can resemble interstitial fluids [14]. Wet-spinning has arisen as an alternative spinning method for the production of microfiber structures with different levels of organization and tunable chemical and physical properties that can enhance cell infiltration and maturation [15]. Additionally, wet-spinning overcomes limitations associated with polymer thermal degradation, which tend to be associated with melt-spinning techniques [16]. Wet-spinning is based on the principle of precipitation during which a phase inversion takes place as the polymer solution is extruded through a spinneret into a coagulation bath [17]. Just as the previous techniques, this too presents limitations, particularly related to the type of polymers that can be used and the need for specialized coagulation baths that may raise the cost of production. Another technique that shares many of the wet-spinning principles is the dry-spinning approach. Here, polymer solidification is easy to achieve by the evaporation of a volatile solvent; contrary to wet-spinning, no coagulation bath is necessary. However, this approach can only be employed to polymers that do not generate viscous melts and that can be processed with volatile compounds. It is a frequent choice to extrude polymers vulnerable to thermal degradation [17].

Among these approaches, electrospinning and wet-spinning are the ones considered most relevant for biomedical uses, particularly for drug delivery systems, since they allow to control fiber production in such a way that complex fiber structures with different organizations and architectures can be attained: (1) side-by-side fibers [18,19], (2) porous [20], (3) helical [21], (4) core-shell [22], (5) hollow [23], (6) tri-axial [24], (6) multilayered [25]. Such morphologies require a precise control of processing parameters. Indeed, polymer spinnability, as well as the fiber porosity and diameter, are dependent not only on the solution properties (e.g., concentration, polymer nature and viscosity) but as well on the system processing parameters (e.g., injection flow rate, coagulation bath, applied voltage) and environment conditions (e.g., temperature and humidity) [24,26]. In the present work, the relationship between processing parameters and fiber morphology, organization, and architecture are analyzed in great detail. The principles of electrospinning and wet-spinning are here emphasized; however, contrary to previous reviews that focused on uniaxial fibers, special attention is given to complex architectures and their main applications in drug delivery and tissue engineering fields. These fiber constructs are gaining more attention each day and, hence, we are here exposing the reasons behind their selection.

## 2. Electrospinning

### 2.1. Principle and Setup

**Figure 1:**
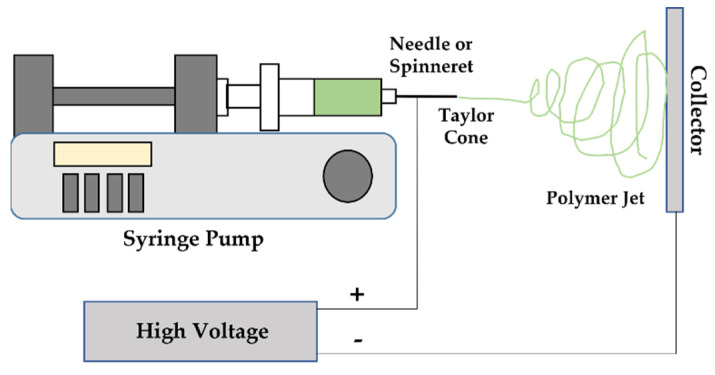
Schematic representation of an electrospinning apparatus.

Electrospinning, a technique that shares characteristics with electrospraying and dry spinning methods, enables the production of fibrous mats with large surface area to volume ratio, controlled pore size and porosity, and tunable mechanical and chemical features [27]. The main components of the eletrospinning setup encompass a high-voltage power supply, a syringe pump, a spinneret and a collector (Figure 1). As the viscoelastic fluid is pumped through the spinneret, a spherical droplet is generated by the confinement of surface tension. Because the droplet is connected to a high-voltage power supply, its surface molecules become charged and start repulsing each other, which then influences the polymeric solution surface tension and destabilizes the pre-existing spherical shape [28]. The applied voltage to the spinneret results in the formation of a Taylor cone, with the ejection of an electrodynamic jet. Once charge repulsion is superior to the surface tension, the droplet deforms into a conical shape, generating a jet at the apex of the cone that initiates a process called “cone-jet” regime. Because of the electric field and the repulsion between surface charges, the jet decreases its diameter and starts to bend. At this point, the “whipping instability” regime sets in, accelerating and fluctuating the jet in a “whipping” movement. The jet diameter rapidly decreases as the solvent is evaporated. Ultimately, micro to nanofibers are generated as the jet reaches the collector and solidifies [29].

Electrospinning is one of the most used techniques in polymer fiber production due to its simplicity, cost efficiency and versatility. Electrospun fibers and/or fiber constructs can be employed in the biomedical and food industries, filtration, energy and even in the fabrication of sensors [27]. In drug delivery systems and tissue engineering, the abilities of electrospun fibers to carry large amounts of bioactive compounds or to be chemically modified to support specific chemical functions are most important and desirable [13,30].

Electrospinning allows for tunable morphologies, porosities and diameters to be generated by controlling/manipulating processing parameters, making this technique attractive for biomimetic fibrous structures and providing new alternatives to restore, maintain and improve tissue functions [31]. A wide range of fiber diameters can be achieved, from few nanometers to less than one micrometer, via this spinning method, which is not always possible using other conventional spinning techniques, such as melt or dry-spinning. Through electrospinning, fibers with long lengths and high surface area are produced [32]. Such technique is also considered more effective and affordable in producing uniform fibrous constructs than techniques such as phase separation, mechanical drawing and melt blowing [33]. Moreover, heat is not necessary during fiber spinning, guaranteeing, this way, structure functionality without material degradation and an enhanced encapsulation efficiency of heat-sensitive bioactive compounds [34]. However, electrospinning still presents several limitations, namely the occasional use of organic solvents, and the low cell infiltration and heterogeneous cell distribution observed in electrospun fibrous mats [35]. Indeed, electrospun structures present high fiber packing density and low porosity, which restrict cell infiltration throughout the innermost regions of the scaffolds, making it difficult to maintain micro-environments that resemble in vivo natural fluid conditions [14].

Fiber diameters, morphologies and textures are dependent on intrinsic features of the polymeric solution—concentration, electrical conductivity, surface tension and viscosity, the distance between the spinneret and collector, injection rate of the spinning solution(s), as well as environmental parameters (temperature and humidity) and the applied voltage [36,37]. Polymeric concentration governs the spinnability of the solution, strongly affecting the fibers morphology. When the solution concentration is increased, the fibers diameter and uniformity are also increased. However, when the concentration overcomes the critical value, the solution can dry inside the needle tip, giving rise to defective, beaded fibers [26,36]. The solution viscosity is another factor influencing the morphology of the fibers. Low viscosities usually result in non-continuous fibers, whereas, at very high viscosities, the ejection rate of the solution through the spinneret can become very difficult [38]. Additionally, the polymer molecular weight not only affects the fibers morphologies but also the electrical and rheological properties, such as surface tension, viscosity, dielectric strength and electrical conductivity [39]. High molecular weight polymeric solutions usually lead to the formation of beadless, large-diameter fibers, whereas low molecular weight polymeric solutions result in beaded fibers [38,40].

Electrical conductivity is another critical parameter in fiber production. The solution’s electrical conductivity is mainly influenced by the type of polymer, solvent used, and the amount of charged ions that can stimulate the formation of a polymeric jet [36,40]. Since a transference of electric charges from the electrode to the spinning droplet is required, low conductive solutions cannot form a Taylor cone. By increasing the conductivity of the solution, fiber jets are subjected to greater tensile forces, forming low diameter non-beaded fibers. On the other hand, conductivity is influenced by polymeric concentration, temperature and polymer, as well as the solvent selected [32]. Jet velocity and transfer rates are affected by the solution injection rate; low rates are desirable to obtain optimal solvent evaporation and, hence, solid uniform fibers. On the contrary, higher injection rates lead to beaded and large diameter fibers, since the time for complete solvent evaporation is insufficient. As a result, control of the tip to collector distance is essential for proper fiber formation [32,38,41]. A suitable distance is required to provide time for liquids to stretch and dry [42]. Shorter collector distances can lead to coarser fiber diameters since the jet is not sufficiently thinned prior to its collection. Yet, by increasing the collector distance, fiber diameters can also be increased [43]. Yet, the choice of a proper solvent is detrimental in this step. Senthil et al. produced poly(styrene-co-acrylonitrile) (SAN) electrospun fibers, applying eight different solvents, which include chloroform (CF), tetrahydrofuran (THF), dimethylformamide (DMF), toluene, 1,2-dichloroethane (DCE), methyl ethyl ketone (MEK), dimethylsulfoxide (DMSO) and ethylacetate (EA). Data showed that the best morphological appearances were achieved with DMSO, DMF and MEK, whereas the solutions dissolved in CF, THF, toluene, DCE and EA were not spinnable. These were also the ones that induced the fastest drying [44]. The type of collector also influences the alignment and morphology of fibers. In research conducted by Prá et al., polycaprolactone (PCL) fibers were produced with the use of three different types of collectors: rotating drum, static copper wires and rotating mandrel. When a rotating drum was used, fibers were stretched by the force of rotational speed, aligning, and reducing fibers diameters. Whereas with the use of a static collector, the electrostatic forces led to fibers being stretched transversely between the wires, resulting in high dense aligned fibers perpendicular to the wires. On the other hand, when a rotating mandrel was applied, electrostatic forces attracted fibers parallelly to the axis and fused fibers were formed transversely to the axis by using high rotational speeds [45]. Furthermore, in a study by Sattary et al., polycaprolactone/gelatin/nano-hydroxyapatite (PCL/Gel/nHA) electrospun scaffolds were placed in two different types of collectors, rotating disc and plate. Results showed that fibers collected on the rotating disc appeared to be more hydrophilic and presented larger pore sizes and faster infiltration of water throughout the scaffold. However, when using the plate, random structures with controlled pore size and lower fiber density were obtained [46].

Solvents surface tension plays a relevant role towards governing the processing efficiency of electrospinning. Usually, with low surface tensions, beadless fibers are formed; yet, in case low electric fields are applied, electrospinning can also take place with low surface tensions [38,40]. One more important parameter consists of the applied voltage that strongly affects the fibers diameters. With high voltages, higher volumes of polymeric solutions are ejected, resulting in large diameter fibers, since higher electrostatic repulsive forces will occur on a jet [47]. More recently, research has also shown that the solution pH can play a major role during fiber production, influencing its properties. In a study by Tam et al., blended lentil flour and hydroxypropyl methylcellulose fibers were produced, in which the pH of the solution altered its viscosity and electrical conductivity. As a result, fibers diameters were also affected [48]. Additionally, in research conducted by Vega-Lugo et al., changes in pH of a poly(ethylene oxide) (PEO) and whey protein isolate (WPI) solution strongly affected fibers morphologies. By using acidic solutions, smooth fibers were obtained, whereas by using neutral solutions, spheres were produced, linked to ultrafine fibers [49]. The deposition angle can also display some effect on the morphology of electrospun fibers. In a study by Al-Hazeem et al., electrospun titanium dioxide (TiO_2_) and polyvinylpyrrolidone (PVP) fibers were produced. Different deposition angles, such as 0°, 45°, 90°, 135° and 180°, were tested, revealing that, depending on the deposition angle, gravity could display either a positive or negative effect [50].

When it comes to the environment parameters, temperature and humidity are the most relevant. By increasing the temperature of the solution, the viscosity decreases, forming fibers with lower diameters [51]. On the other hand, when humidity is increased, the number of pores increases, influencing pore distribution and solvent evaporation rate. With low humidity, the solvent is evaporated faster, causing clogging of the needle tip [40].

### 2.2. Categories

The electrospinning technique can be classified into five main categories: (1) blend electrospinning, (2) co-axial electrospinning (within which it is also possible to identify the tri-axial electrospinning), (3) emulsion electrospinning, (4) melt electrospinning and (5) gas jet electrospinning [52].

The blend electrospinning approach consists in preparing a polymeric solution in which a payload (i.e., bioactive molecule, drug, etc.) is introduced prior to electrospinning. Usually, the payload is homogenized in a polymeric matrix, which encapsulates and protects it. Here, the payload becomes less accessible, since it is not only present at the surface of the fibers but as well at the interior, which can allow a more prolonged release profile. This approach has been applied for different active substances, including antibiotics, cytostatic and anti-inflammatory drugs, antimicrobial peptides, etc. [53]. This approach is considered the conventional electrospinning approach because of its simplicity. However, it should be noted that the solvents used to disperse the bioactive molecules within the polymer matrix, if not compatible, can lead to the loss of biological activity or even charge, directing biomolecules to migrate along the jet surface and exposing them instead of encapsulating them, thus reducing their protection [54].

In the co-axial approach, a spinneret with two concentric nozzles is connected to an high-voltage power supply and fed by two distinct solutions to generate a core-shell morphology [54]. In drug delivery systems, it is common for the biomolecules or drugs to be introduced via the inner jet and form the core, and for the outer jet to be made of a protective polymeric layer that works as a diffusion barrier [55,56]. The cargo can be introduced at the core on its own or blended with other polymeric materials; still, when formed, the core and shell layers are fully separated [13,55]. Core-shell fiber systems have already been designed to carry anti-inflammatory, antimicrobial, anticancer and analgesic drugs, including acetaminophen, ibuprofen, amoxicillin, curcumin, ampicillin, ciprofloxacin, doxorubicin and paclitaxel [57,58,59]. He et al., for instance, proposed a modified co-axial electrospinning method for a biphasic drug delivery system. The core solutions were constituted by polymer ethyl cellulose (EC) and drug ibuprofen (IBU), whereas the shell solutions contained IBU and polyethylene glycol (PEG). Results showed that the core solutions successfully enabled the formation of spindles-on-a-string (SOS) hybrid structures and a biphasic release profile was achieved, with 40% release during the first hour and a sustained release of the remaining IBU [60]. Tri-axial electrospinning is a more complex variation of the co-axial approach. It can also be employed to produce new fibrous architectures by using a main power supply, a tri-axial spinneret, collector, and three individual pumps that feed the system with three individual solutions, giving rise to fibers with three distinct layers. This way, a different method for the production of materials with desired functional performances and expanded applications for drug delivery systems can be achieved [61]. One of the main advantages of tri-axial electrospinning is that the originated fibers contain a blend of the inherent features of the solutions that feed the system, making possible the use of non-electrospinnable polymers that, on their own, could not generate fibers, thus solving problems related with possible incompatibilities between drugs and organic solvents. In addition, with tri-axial electrospinning, it is possible to manipulate the continuous fabrication of core-shell fibrous structures to facilitate loading of poor-soluble drugs and, thus, reach sustained release profiles [61,62].

The emulsion electrospinning approach is based on the principle of multiple phases within a spinning solution, in which homogeneous blending does not occur. The spinning solution is thus formed of a liquid or continuous phase, forming the “shell” of the fiber, and a droplet phase that will give rise to the core of the fiber [54]. The emulsion electrospinning requires a similar setup to the blend electrospinning approach; however, in the first two immiscible solvents or solution components are spun simultaneously. In this method, bioactive molecules and surfactants form water/oil emulsions that are then added to the polymer matrix [63]. Afterwards, as fiber trajectory shapes up, emulsion droplets are stretched into an elliptical shape and the continuous phase solvent is evaporated, leading to a difference in the viscosity gradient, which guides the core material to settle within the fiber matrix instead of at the surface [64]. According to Nikmaram et al., electrospun fibers produced by this method have gained considerable attention as vehicles for sustained and controlled release of specialized payloads [63].

In the melt electrospinning category, heat transfer and quenching of the melted jet is performed, rather than mass transfer and evaporation of the solvent (as happened in the previous approaches). As a result, micrometric fibers suitable for biomedical, tissue engineering (supporting cell attachment, proliferation, and infiltration), energy and environmental purposes are obtained [65,66]. Gas jet electrospinning results from an upgrade on the melt electrospinning approach. One major limitation of melt electrospinning is the need to control the polymer melt temperature, requiring several heating zones along the apparatus to guarantee an even distribution of temperature. Here, however, only an additional gas jet device is attached to the conventional electrospinning setup. Through gas jet electrospinning, the polymer jet is involved in a heated gaseous atmosphere that guarantees a completely uniform distribution of heat throughout the polymer jet at the nozzle, delaying polymer solidification. By increasing gas flow rate, higher drag forces on the jet surface can take place, resulting in very thin fibers [52].

In more recent years, wet-electrospinning has been identified as a modified-electrospinning version that combines principles of electrospinning with those of wet-spinning. In such a method, the metallic collector is replaced by a liquid bath. This method allows for fibrous materials to be generated without extra chemical additives, being commonly applied in tissue engineering. This approach was reported by Wang and Ziegler et al., in which starch-based electrospun fibers were obtained. Results indicated that the use of sodium palmitate increased amylose water stability at room temperature and the solution conductivity. In addition, pullulan increased the fibers molecular entanglement [67]. In a different study, co-axial wet-electrospinning was applied, producing core-shell starch-hyaluronic acid (HA)/polyurethane (PU) nanofibers, with starch and HA at the outer layer and PU at the core. The outer layer benefited from the collector bath by increasing the surface hydrophilicity, biocompatibility and biodegradability, whereas the inner layer guaranteed the mechanical durability of the scaffold [68].

### 2.3. Fiber Structural Organization

**Figure 2:**
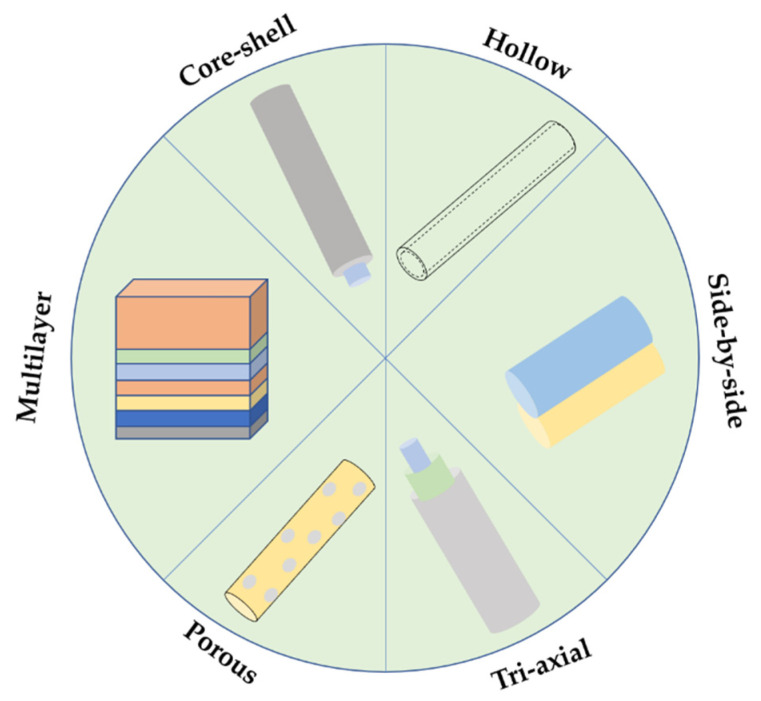
Schematic representation of the most common electrospun fiber structural organizations.

Several fiber structural organizations can be obtained using designed spinnerets, including hollow, core-shell, tri-axial, porous, side-by-side and multilayer (Figure 2). By controlling the solution and processing parameters, such as fiber body size, mass and content, fibers with active surface properties can be generated with impactful architectures in nanofluidics, drug delivery, nano-supports, energy storage units, gas sensors, etc. [26,69].

#### 2.3.1. Core-Shell

**Figure 3:**
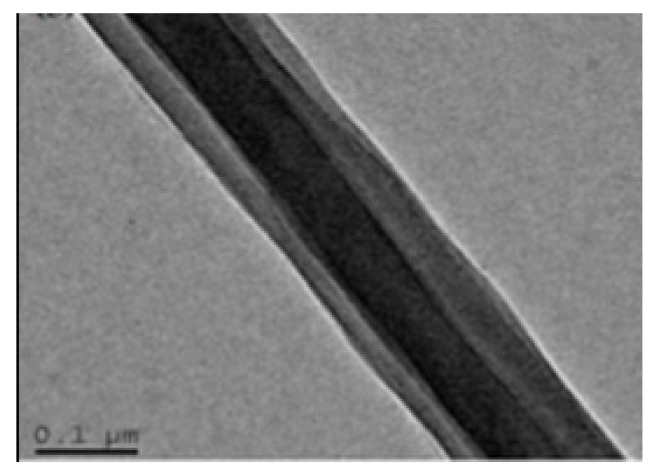
SEM micrograph of a core-shell electrospun fiber (adapted from [80], published by MDPI, 2019).

Core-shell fibers (Figure 3) keep the intrinsic properties of the nanofibers at the core and impart some of those features onto the shell. The co-axial spinneret is frequently applied to induce this structural organization. In many instances, bioactive molecules are employed as functional agents at the shell for an easier access, living the structural integrity of the fiber to be maintained by the core [22,70]. In others, even more frequent, the opposite occurs, with the shell working as a protective layer that guarantees a more programmed and spaced liberation of the active agents. For instance, Mahdieh et al. loaded silver nanoparticles (Ag NPs) at the core of a fiber in which the shell was composed of polycaprolactone (PCL), polyethylene glycol (PEG) and zinc oxide nanoparticles (ZnO NPs). Overtime, liberation of Ag NPs was evidenced via pore formation throughout the shell, which did not compromise the mechanical stability of the fibers [27]. In another study by Pedersbæk et al., core-shell fibers were designed with PCL and tetracycline hydrochloride (TCH) at the core and blends of PCL/polylactic acid (PLA) at the shell. Data showed that it was possible to regulate the TCH release profile by altering the polymer ratio at the shell [13]. Indeed, by modifying the type of polymer, the blend of simply the polymer ratio a completely different drug delivery system is attained. This allows for a completely different set of applications to be envisaged via co-axial electrospinning. For instance, Abdelhakim et al. engineered a taste-masked system for the development of a palatable oral film for children. In that system, taste-masking polymers, Kollicoat^®^ Smartseal (KCT) and Eudragit^®^ (E-PO) were used. Core-shell electrospun fibers were proven more effective towards taste-masking, in comparison with fibers using either polymer separately. By injecting E-PO in the shell solution, drug release can occur more effective for absorption throughout the gastrointestinal tract [71]. Core-shell fibers have also been used as nanodrug containers to load natural-origin biomolecules, such as curcumin (Cur) and generated effective sustained release drug delivery systems [72].

Many studies have reported on the importance of adjusting and optimizing electrospinning parameters for guaranteeing the stability and structural integrity of complex systems such as the core-shell. According to Mao et al., the viscosity of the shell solution should be high enough so that the viscous stress imparted by it on the core overcomes the interfacial tension between both solutions, giving rise to a Taylor cone and, consequently, a polymer jet [73,74]. The shell polymer-solvent system has to be highly electrospinnable to allow the formation of a core-shell structure [75]. In these systems, the viscosity and electrospinnability of the core is not as critical as the shell material, yet it should guarantee the formation of a continuous fiber [22]. The shell material can also prevent jet break-up of the core by (1) strain hardening the interface via rapid stretching during whipping or by (2) reducing the surface forces acting on the core solution, which is dependent on the polymer selection [74].

Similar to the conventional electrospinning, in the co-axial approach, an increase in the solution concentration leads to an increase in the fiber diameter caused by the higher amount of material in the jet. Additionally, the increase in the core diameter can lead to a decrease in the sheath thickness, since the shell polymer needs to be distributed over a larger surface area [76]. The nature and functionality of the solvent used in the core solution plays an important role on the morphology of the resulting core-shell structure. According to Li et al., a high vapor pressure solvent in the core can lead to a thin layer at the core and sheath interface due to rapid evaporation [77]. The layer traps the solvent that diffuses more slowly; when it leaves the solidified structure, a vacuum is created, causing the core structure to collapse under pressure. Another extremely important parameter is the solution conductivity. Generally, solutions with high conductivity presents higher surface charge density, increasing the elongational force on the jet caused by self-repulsion of the excess charges under an electrical field [78].

Flow rates have been reported to exert a direct control over the dimensions of the core and shell layers. When the shell flow rate is constant, a range of core flow rates must be considered; stabilized, regular Taylor cones can be generated to attain core-shell configurations. When the core flow rate is too low, a small amount of solution is delivered; therefore, it is not possible to reach a continuous incorporation of the core into the shell. Yet, when the core flow rate is too high, the size of the core Taylor cone increases so that the viscous drag applied by the shell solution does not confine the core solution within the Taylor cone, causing the inner cone to lose its shape and mix the inner and outer polymers, forming a blend [74]. As a norm, the core flow rate should always be smaller than the shell flow rate, so a complete coverage of the core can be attained and continuous fibers can be formed [22]. Morais et al. showed exactly that by generating core-shell fibers with smooth surfaces without irregularities, which indicates that the implant coating surface has an extensive pore network and tubules that completely cover the inner region for a sustainable release of the entrapped drug [79].

#### 2.3.2. Tri-Axial

Tri-axial fibers are made of three layers, in which limitations related to solvent incompatibilities or drug availability can be quickly solved by strategical positioning within the layers. Such constructs are frequently used in place of co-axial systems to improve the solubility of poor soluble drugs and, thus, to reach a sustainable drug release [61,62]. An innovative tri-axial electrospun system has been engineered based on cellulose acetate (CA) loaded with a model drug, the metformin hydrochloride (MET). Tri-axial fibers were successfully produced using different organic solvents for preparing each layer, exhibiting a linear morphology; no drug loss was detected throughout the process. A fine sustained drug release profile was observed, which attested to the potential of the system for the release of water-soluble drugs [72]. Similarly, Ding et al. employed the tri-axial electrospinning method to generate fibers composed of Eudragit^®^ S100 polymer and aspirin, once again guaranteeing a sustained drug release throughout time [81]. Bai et al. and Kang et al. also used a tri-axial electrospinning method to improve the solubility of poor water-soluble drugs. In both studies, polyvinylpyrrolidone was used for the production of fibers loaded with ibuprofen and ketoprofen, respectively [82,83]. In a report by Wang et al., drug reservoirs were produced, being composed of tri-layer nano depots. Tri-layer depots of CA and acyclovir (ACY) were prepared, as well as a core-shell fiber matrix with a blank polymer coating on a drug-loaded core component. Results showed tri-layered fibers eliminated potential initial burst releases and prolonged the release time, also improving drug diffusion mechanisms [62].

Similar to the core-shell approach, here too studies have been published on the influence of processing parameters in the fibers’ architecture. For instance, Xu et al. demonstrated that the selection of solvent for the different layers requires an adjustment of specific flow rates, so smooth morphologies can be attained. Further, production of larger core sections should be targeted so that efficient drug loading is achieved; this can be attained by increasing the viscosity of the core solutions [72]. The effect of solvent volatilities and polymer molecular weights on tri-axial electrospun fibers has also been evaluated. Stable jets have been shown to only be attained when the molecular weight of the intermediate polymer is equal or slightly smaller than the polymer constituting the outer layer. Using a core made of a mineral oil was also seen to not alter the fibers diameters, if the processing parameters remain unchanged, compared to a hollow scenario. As the humidity raised above 30%, pores were formed on the outerlayer of the tri-axial fibers, increasing the surface area, and consequently improving oil permeation throughout the layers and subsequent release [84].

#### 2.3.3. Hollow

**Figure 4:**
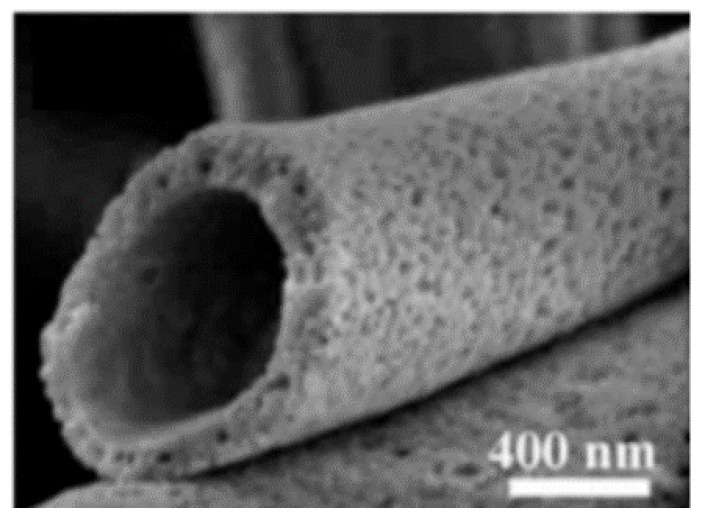
Scanning electron microscope (SEM) micrograph of an hollow nanofiber (adapted from [80], published by MDPI, 2019).

Hollow electrospun fibers (Figure 4) have been gaining considerable attention for their unique properties, including extensive surface area, larger accessible active area, high porosity and sensitivity, which make them exceptional for surface-related applications, such as chemical sensors, photocatalysis and electromagnetic wave absorbing materials, and health- and environment-related devices [85]. Indeed, the surface area of these fibers has been reported as two times larger than conventional fibers [85,86]. Hollow fibers can be produced by two methods: chemical vapor deposition (CVD) or direct co-axial electrospinning. In the CVD method, the precursor polymer (core) processed in the form of nanofibers by conventional electrospinning is coated with other polymers or metals. Then, the template material (core) is dissolved and the hollow fibers are dried via centrifugal rotation dryers or calcining in furnaces [87]. In the co-axial spinning method, fibers are produced similarly to core-shell nanofibers. However, a dissolution of the core material with a selective solvent takes place at the end of the process [88].

Hollow nanofibers can be filled with different substances depending on the application envisaged and their stability is reliant on the relationship established between the many processing and solutions parameters [89]. In a study by Lee et al., hollow carbon nanofibers (HCNFs) were produced by co-axial electrospinning, using poly (styrene-co-acrylonitrile) (SAN) and poly (acrylonitrile) (PAN) solutions. Solutions concentration and flow rate determined the outer diameters and the wall thickness of the nanofibers. Here, SAN displayed a good thermal sustainability that prevented the shrinkage of the PAN shell [90]. Porous hollow nanofibers have also been produced, using silicon oil as the core material and polymethyl methacrylate (PMMA) and polycarbonate (PC) as the shell materials. Again, solvents and polymer concentrations exhibited a great influence on the diameter and thickness of the hollow fibers. Indeed, the diameter of the fibers was reduced by resorting to solvents with high dielectric constants, while increasing the polymer molecular weight allowed for the wall thickness and pore size to be enhanced, even though the overall surface area decreased [91].

In these hollow structures, upper and lower limit concentrations must be established. Lee et al. studied the effect of PMMA solution concentration on PMMA/PC fibers’ spinnability and morphology and determined that, by using concentrations inferior to 7% *w*/*v,* fiber production was unviable. However, at a concentration of 15% *w*/*v*, spindle-like hollow fibers were formed. The increase of PMMA concentration allowed for the formation of hollow fibers with increased diameters. Still, it was with a concentration of 20% *w*/*v* that uniform hollow fibers were attained [91]. Furthermore, the dielectric constants of the solvent displayed relevant effects on the morphology and diameter of the resulting fibers; high dielectric constant solvents decreased the formation of beads and the diameter of the fibers. The reduction of fibers diameters was most likely due to a high repulsive force present in the thin jet that led to a phenomenon known as splitting, which caused further thinning of the jet. By increasing the viscosity and molecular weight of PC, fine hollow fibers were generated. The thickness of the produced hollow fibers and their overall average diameters increased when the molecular weight of PC increased. The differences on the diameters could be the result of an entanglement effect between different molecular weight polymers [91].

#### 2.3.4. Porous

**Figure 5:**
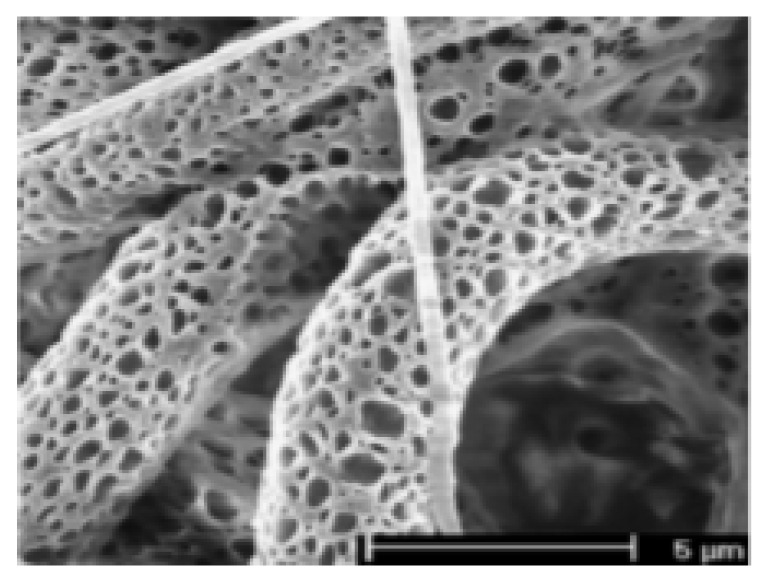
SEM micrograph of a porous electrospun fiber (adapted from [80], published by MDPI, 2019).

Porous fibers (Figure 5) can be applied in a large variety of fields, such as filtration, fuel cells, filtration membranes, catalysis, tissue engineering, and drug delivery [20]. Porous fibers can be produced with a special topology, using specific solvents or solvent mixtures under controlled media. In one option, the most common, immiscible components are electrospun and one of the polymers is then dissolved, generating randomly located pores; this approach is based on a phase separation strategy in which polymers may present different evaporation rates. Within the phase separation strategy, it is possible to distinguish other methodologies, namely vapor-induced phase separation (VIPS), nonsolvent-induced phase separation (NIPS) and thermally-induced phase separation (TIPS). Additionally, selective dissolution, rapid phase separation and selective pyrolyzate composite formation may also be proposed to originate pores along fibrous mats’ structure [92]. PLA/PVP porous nanofibers have been produced by VIPS with a controllable porosity density via the amount and ratio between two polymers [93]. Similarly, polylactide (PLLA) and polylactide-co-poly(ε-caprolactone) (poly(LLA-co-CL)) fibers have been electrospun into tunable porous fibers by varying the lactide/caprolactone ratios. Data demonstrated that porosity can be tunable by adjusting segments content and changing solvents [94]. Moreover, PCL porous fibers were produced using binary solvent systems, which included chloroform (CHF), dichloromethane (DCM), tetrahydrofuran (THF) and formic acid (FA), mixed with dimethyl sulfoxide (DMSO), and revealed that high solvent ratios can enhance pore formation. To the contrary, by using low solvent ratios, a more pronounced ribbon cross section of the fibers is obtained [95]. The NIPS technique has been used to produce porous and hollow PAN fibers [96]. The solvent/nonsolvent exchange occurs and a porous structure is formed when the polymer is immersed in a nonsolvent bath. The specific surface area of PAN was higher, in comparison with the traditional technique [97]. In a different study, porous PAN fibers were produced by electrospinning, using a ternary system of PAN/N, N-dimethylformamide (DMF)/water. In this case, the fibers diameter was increased by increasing the surface tension and viscosity of the PAN solution. In addition, the surface area of the porous PAN nanofibers was larger, compared with the nonporous PAN nanofiber formed under equal conditions [98]. The effect of the polymer concentration on the spinnability and morphology of the fibers was also evaluated by Yu et al. Here, whenever the PAN concentration was increased to values above 5% *w*/*v*, better spinnability was achieved and beadless mats were acquired. In addition, the fibers diameters became more uniform and increased by increasing the polymer concentration. The surface of the fibers was smooth without pores. However, by introducing water in the polymer solution, the morphology of the fibers altered; rougher fiber surfaces were obtained using 5–8% *w*/*v* PAN solution with an increase of water content [98].

The morphology and structure of the mats (pore size, depth, shape, and distribution) can also be influenced by the collector temperature, as demonstrated by Kim et al. [99]. Microscopical observations showed that, at around 21 °C, small pores can be formed on the surface of the fiber. However, by increasing the collector temperature to 40 °C, the pore size also increased. This occurrence was due to the solvent boiling point; during the electrospinning process, the solvent evaporates when it goes from the tip of the syringe to the collector. Nonetheless, by heating the collector to temperatures equivalent to the solvent boiling point, the residual solvents entrapped inside the fiber were evaporated. Therefore, the evaporation of the solvent can lead to the formation of pores with larger diameters [99].

#### 2.3.5. Side-by-Side

In the side-by-side approach the capillaries stay beside each other; two polymeric solutions enter in physical contact at the end of the spinneret tip. Fibers produced using this method possess features from both extruding polymers. Several studies on side-by-side fibers have been conducted. For example, Liu et al., using camphric acid (CSA), polyaniline (PANI) and polyethylene oxide (PEO), demonstrated that side-by-side electrospinning can overcome the poor spinnability of PANI by loading it into PEO fibers that were being extruded at the proximity. Fibers produced via this approach presented higher fiber strength, elongation at break and superior ductility and electrical resistivity, in comparison with blended electrospun fibers [18]. Synergistic phenomena have also been observed with polyvinylidene fluoride/polyimide (PVDF/PI) side-by-side fibers, in which enhanced mechanical strength and thermal stability, over individual polymers or blends, was attained [19].

As with the core-shell structure, electrospinning parameters play an important role in the production of side-by-side configuration fibers. PAN and polyurethane (PU) have been used in these bicomponent systems, generating curly fibers. This morphology resulted from shrinkage within the fibers, with one component compressing the adjacent. Yet, by increasing PAN concentration and flow rate, the predominance of curly nanofibers was reduced. To the contrary, when PU concentration increased, more curly nanofibers were formed [100]. According to Chen et al., curly nanofibers function as nanosprings, influencing the mechanical properties of the resulting mat. They demonstrated that poly(m-phenylene isophthalamide) and thermoplastic polyurethane (TPU) can generate aligned nanospring fibers endowed with higher elongations and higher toughness than nanofibrous mats without nanospring [101]. The side-by-side approach can also be employed to improve the photocatalystic and electrochemical properties of polymers by incorporating oxides within metal-based fibers [102] or by generating mixtures that are different from actual blends, in which conductive polymers are presented in an interconnected way [103], respectively.

#### 2.3.6. Multilayered

In general, multilayered electrospun fibrous constructs are composed of several layers in which each layer is fabricated with a specific pore size and surface properties. The first layer is usually produced to perform pre-filtration and provide high mechanical strength during high flux filtration, whereas, pore size and the selectivity of adsorption are considered in the consecutive layers, which are designed to respond to the demands of the desired application [25,104]. Deposition/coating of the functionalized nanomaterials on the mid or top layers aims at an efficient adsorption of contaminants, including bacteria, fungi, viruses, heavy metal ions, dyes and toxins [105]. Even though multilayered systems are highly regarded in many fields, one obstacle remains: the weak mechanical performance of the layered system, raising the risk of splitting [106].

Multilayered scaffolds have been produced with aligned, unaligned, dense, and porous fiber organizations. They can be employed in the repair of flat and tubular tissue layers in which various cell types can be seeded and grow prior to implantation [107]. For instance, Chainani et al. constructed a multilayered PCL scaffold in which tendon-derived extracellular matrix (TDM) cells were coated. Yield strain was increased and cell infiltration throughout the scaffold showed enhanced expression of type I collagen over time [108].

Processing parameters, including applied voltage, feed rate and air gap, and solution parameters, such as concentration, viscosity and polymer molecular weight, as well as environmental parameters, such as temperature and humidity, strongly influence the spinnability of solutions and the resultant fiber characteristics [109]. Ma et al. showed that, with a membrane porosity of 80% volume, the pore size of the membrane had a correlation with the electrospun fibers diameter. The average pore size was around 1–3 times higher than the average fiber diameter and the highest pore size was 2–10 times higher than the average fiber diameter. By altering specific processing parameters, such as the solution and operation parameters, different nanofiber diameters and pore sizes were obtained [110]. Doping nanofibers of cellulose acetate (CA) with different concentrations of cellulose nanocrystals (CNC) was proven to enhance the mechanical properties, surface area, hydrophilicity and filtration performance of the multilayered system (all equal layers) as CNC incorporation rose [111]. Bio-based three-layered electrospun mats of poly(3- hydroxybutyrate-co-3-hydroxyvalerate) (PHBV), CA and chitosan were also generated to improve the rejection ratio of a filtration membrane. Here too, the increasing amounts of chitosan free groups enhanced the membrane efficiency, improving the absorption of metal ions from water [112].

### 2.4. Tissue Engineering and Drug Delivery Applications

For many years, biomedical researchers have fabricated multidisciplinary platforms to mimic natural-origin features of cellular tissues. In fact, various tissue injuries have been treated and healed by mimicking physiological microenvironment [113]. Drugs can also be incorporated into electrospun fibers by dissolving and mixing the drug into the electrospinning solution for a controlled release of active molecules. When electrospun fibers degrade or swell, the drug inside the fibers is released [114,115,116]. Nonetheless, the biocompatibility and toxicity of the encapsulated compounds need to be addressed, with major challenges that include in vivo instability and low bioavailability requiring further fiber processing optimizations [117].

In Table 1, examples of the previously listed structural organizations and respective processing parameters, with significant biomedical outcomes, have been provided. Data has been collected from research published in the past five years.

**Table 1:** Main applications of electrospun fibers with various structural organizations and respective solution/processing parameters.

	Active Agents						
Polymers	Name	Characteristics	Structural Organization	Solution and Processing Parameters	Major Findings	Envisaged Applications	Ref.
PU; HA/St	HA	Group of polysaccharide molecules, usually found on connective tissues	Core-shell	Core: PU (12% *w*/*v*) was dissolved in DMF; the solution was injected at 675 mL/min through a co-axial needle with inner diameter of 1.6 mm; Shell: both St (9% *w*/*v*) and HA (1% *w*/*v*) were dissolved in water; the solution was injected at a feed rate < 0.135 mL/min through a co-axial needle with outer diameter of 2.0 mm.	A uniform structure was obtained; modification with HA enhanced cell adhesion into fibrous scaffolds	Skin scaffolding systems; wound healing	[118]
PCL; PEG	Ag NPs; ZnO NPs	AgNPs display unique optical, electrical, thermal, and biological properties, being used for several antimicrobial and medical-coating applications; ZnO is an essential ingredient for several enzymes, being used for pain relieve and as an antimicrobial agent.	Core-shell	Core: Ag NP (0.01/0.02% *w*/*v*) were diluted in water and the solution was injected at 0.0067 mL/min; Shell: PCL (14% *w*/*v*), PEG (7% *w*/*v*) and ZnO NP (1.6% *w*/*v*) suspensions were prepared separately in CHF/DMF (17:10); the shell solution was injected at 0.0167 mL/min; Voltages of 16/20/21 kV were applied; the spinneret-collector distance was kept at 20 cm and the co-axial spinneret presented a 0.90 mm inner diameter and 1.60 mm outer diameter.	Ag NPs showed a fine-tuned release rate through pores formed along the shell structure; fibers presented excellent mechanical stability	Drug delivery systems	[27]
PLA; PCL	TCH	Bacteriostatic agent that inhibits protein synthesis; effective antibacterial agent.	Core-shell	Core: PCL (10% *w*/*v*) dissolved in CHF; Shell: PLA (10% *w*/*v*) in CHF; TCH (5% *w*/*v*) was dissolved in methanol and then added to the PLA solution (CHF:methanol ratio of 19:1); Voltages of 12.5–17.0 kV were applied and a needle with a 2.5 mm outer diameter was used.	The composition of the shell influenced the initial burst release, by working as a diffusion barrier	Drug delivery systems	[13]
PCL; PGS	Heparin	Polyanionic polysaccharide; works as an anticoagulant.	Core-shell	Core: PGS (0/40/60/80% *w/v*) was dissolved in TFE; the solution was injected at 0.030 mL/min; Shell: PCL (13% *w/v*) was dissolved in TFE; the solution was injected at 0.180 mL/min; A voltage of 15 kV was applied; the spinneret-collector distance was of 15 cm and the needle presented an inner diameter of 0.94 mm and outer diameter of 2.50 mm.	Slow degradation of PCL provided the fibers with structural integrity, whereas fast degradation of PGS increased their elasticity; addition of PGS and grafting of heparin enhanced the attachment and proliferation of human umbilical vein endothelial cells	Tissue engineering scaffolds	[119]
PCL	ShHL	Derived from *Halomonas levan*; is a bacterial-origin linear polymer that possesses anti-oxidant and anti-cancer activities.	Core-shell	Core: PCL (10% *w*/*v*) was dissolved in THF and DMF (1:1); the solution was injected through a needle with an inner diameter of 1.3 mm; Shell: ShHL (7% *w*/*v*) was dissolved in water; The solution was injected through a needle with an outer diameter of 2.7 mm.	The increase of ShHL content led to higher ultimate tensile strengths; fibers showed high potential in decreasing neointimal proliferation and thrombogenicity of grafts and prosthesis	Tissue engineering scaffolds; blood-contacting devices	[120]
PCL; PLGA; GN	RhB; FITC	FITC is a derivative of fluorescein, used for flow cytometry detection.	Tri-axial	Core: PCL 1% *w*/*v* dissolved in HFP, injected at 0.00833 mL/min; Intermediate layer: GN 2% *w*/*v* dissolved in HFP, injected at 0.00833 mL/min; Shell: PLGA 25% *w*/*v* dissolved in HFP, injected at 0.0125 mL/min; 0.25% *w*/*v* RhB and 1% *w*/*v* FITC were used as active agents; A 0.75–1.5 kV voltage was applied, using a collector distance of 10–25 cm.	The addition of PCL increased the fibers elastic modulus; fibers showed ideal support for the growth of mesenchymal stem cells	Regenerative engineering and drug delivery systems	[121]
CA; PVP	KET	Nonsteroidal anti-inflammatory drug, used to treat pain and/or inflammation cause by arthritis.	Tri-axial	Core: CA/KET, injected at 0.0167 mL/min; Intermediate layer: bank CA layer, injected at 0.00833 mL/min; Shell: PVP/KET, injected at 0.0167 mL/min; A voltage of 17 kV was applied, with a collector distance of 20 cm.	Fibers presented good dual drug release, with more accurate release contents at the initial stage and more prolonged sustained release at the second stage	Drug delivery systems	[122]
PLA; PCL	DOX	Chemotherapy medication.	Porous	PLA and PCL were dissolved in DCM/DMF (98:2) in ratios of 3/1, 1/1 and 1/3, with a total polymer concentration of 8% *w/v*; CuS NPs were synthesized in a mixture of CuCl_2_·2H_2_O, sodium citrate and Na_2_S and then added to the PLA/PCL mixture; the solution was injected at 0.0333 mL/min using a voltage of 15 kV.	Fiber membranes promoted cutaneous wound healing, along with enhanced mechanical support and controlled release of therapeutic copper ions	Drug delivery systems; wound healing	[123]
PCL	CAM	Antibiotic used to treat eye infections.	Porous	CAM (4% *w*/*v*) was added to the electrospinning solution after PCL (12.5/15% *w*/*v*) was dissolved in mixtures of acetone, CHF, DCM, DMSO, THF, acetic acid and formic acid; a blunt metal needle with 0.60 mm diameter was used; the solution was injected at 0.0167 mL/min; the spinneret-collector distance was kept at 15 cm and voltages of 11, 13 and 15 kV were applied.	Drug release from porous microfibers was facilitated; changes in humidity allows for fiber structure to be tuned and, consequently, the drug release profile	Drug delivery systems	[124]
PLLA	-	-	Porous	PLLA (8% *w*/*v*) was dissolved in CHF at room temperature; SLES (25% *w*/*v*) was then added to the PLLA solution; the solution was injected at 0.0083 mL/min; a metal needle of 0.7 mm diameter was used; 6 kV were applied, and the distance of the spinneret-collector was of 2.0 cm.	3D mats were formed with porous fibers and the addition of SLES surfactant led to higher crystallinity degree and enhanced cell proliferation	Tissue engineering scaffolds	[125]
SF; PLLA	-	-	Side-by-side	Side 1: SF (10% *w*/*v*) was dissolved in HFIP; Side 2: PLLA (4% *w*/*v*) was dissolved in HFIP; Each solution was injected at 0.0055 mL/min; 15 kV voltage was applied, and the spinneret-collector distance was kept at 15 cm.	Results showed a dependence of the molecular orientation and secondary structure of the fibers on the alignment and annealing conditions; fibers treated with methanol and heated at 80 ºC revealed enhanced mechanical features	Medicine regenerative scaffolds; drug delivery systems	[126]
PVP; PAN	DXM; 1,8-naphthalene anhydride; PMI	DXM is a corticosteroid, similar to natural hormones produced by adrenal glands; PMI is an anhydride diester, that can also be used as an intermediate for the synthesis of perylene carboxylic derivatives.	Side-by-side	Side 1: PVP (15% *w*/*v*) was dissolved in DMF; DXM and 1,8-naphthalene anhydride were added to the PVP solution; Side 2: PAN (8% *w*/*v*) was dissolved in DMF; DXM and PMI were added to the PAN solution Voltages of 17–20 kV were applied; each solution was injected at 0.00835 mL/min; the spinneret-collector distance was of 15 cm.	Self-supporting properties were exhibited when PVP was dissolved in water; ideal biphasic drug release profiles were attained	Biphasic drug release	[127]
PVP; EC	KET	Nonsteroidal anti-inflammatory drug, used to treat pain and/or inflammation cause by arthritis.	Side-by-side	Side 1: PVP (8% w(v) and KET (2% *w*/*v*) were both dissolved in ethanol; Side 2: EC (24% *w*/*v*) and KET (2% *w*/*v*) were both dissolved in ethanol; A voltage of 12 kV was applied; a spinneret-collector distance of 20 cm was used, and solutions were injected at 0.0167 mL/min.	PVP dissolved very rapidly and delivered a loading dose of ketoprofen, whereas EC released ketoprofen in a more sustained way; when PVP was added to EC, the second stage of release was accelerated	Drug delivery systems	[128]
Alginate; PCL; PEO	ZnO NPs; Triton X-100	Triton X-100 is a common nonionic surfactant, with conductive and dissipative properties.	Multilayered	Layer 1: PCL (10/20/30% *w*/*v*) was dissolved in GAA/Ac (1/1 and 3/1); a spinneret-collector distance of 20 cm was applied; 15 kV voltage were applied, using a 0.4 mm diameter needle; the solution was injected at 0.0167 mL/min Layer 2: SA (1% *w*/*v*) was dissolved in a water suspension containing 0.25% *w*/*v* ZnO NPs; PEO powder and Triton X-100 were added; a spinneret-collector distance of 15 cm was applied; 12.5 kV voltage were applied, using a 0.4 mm diameter needle; the solution was injected at 0.0125 mL/min	PCL provided good mechanical properties to the membrane, and worked as a protection from the external environment; alginate internal layer promoted cell viability, removed exudates, and allowed gas exchanges; ZnO NPs was antibacterial and bacteriostatic	Skin wound patch	[129]
PCL; PLGA	RhB	RhB is an organic compound and a dye, used within water to determine direction flow.	Multilayered	Layer 1: PCL (10% *w*/*v*) was dissolved in DCM-DMF (80:20); the solution was injected at 0.0334 mL/min; 20 kV of voltage were applied; a 1 mm diameter needle was used; a spinneret-collector distance of 10 cm was used Layer 2: PLGA (24% *w*/*v*) was dissolved in DMF; RhB (5% *w*/*v*) was added to PLGA solution in a ratio of 65:35; the solution was injected at 0.0501 mL/min; 20 kV of voltage were applied; a 1 mm diameter needle was used; a spinneret-collector distance of 10 cm was employed Layer 3: similar to Layer 1	A prolonged release was achieved; FE and computational models could both provide accurate predictions of drug release	Prolonged drug delivery systems	[130]
PLLA	-	-	Multilayered	Layer 1: PLLA (7.5% *w*/*v*) was dissolved in HFIP; the solution was injected at 0.0167 mL/min; a 0.8 mm diameter needle was used; 12 kV voltage were applied and a spinneret-collector distance of 15 cm was kept Layers 2 and 3: similar to Layer 1	Multilayer structures presented higher tensile strengths and favored the colonization and migration of H9C2 cells	Tissue regeneration scaffolding systems	[131]
PCL; mGLT	-	-	Multilayered	Layer 1: PCL particles were dissolved in 18% *w*/*v* TFE; a voltage of 8 kV was applied Layer 2: mGLT (20% *w*/*v*) was dissolved in 95% *w*/*v* TFE; 15 kV voltage were applied Both layers were alternated, and in each layer a 22 G needle was used, with a spinneret-collector distance of 15 cm; both solutions were injected at 0.0334 mL/min	mGLT uniform distribution was attained and the scaffold maintained its mechanical strength; photocrosslinking allowed to form multilayered constructs, mimicking the structure of native tendon tissues	Tissue and ligament regeneration	[132]

Abbreviations: PU—polyurethane; HA—hyaluronic acid; St—starch; DMF—N,N-dimethylformamide; PCL—polycaprolactone; PEG—polyethylene glycol; AgNPs—silver nanoparticles; ZnO NPs—zinc oxide nanoparticles; CHF—chloroform; G—gauge; PLA—poly-lactic acid; TCH—tetracycline hydrochloride; PGS—poly(glycerol sebacate); TFE—trifluoroethanol; ShHL—sulfated hydrolyzed *Halomonas*; THF—tetrahydrofuran; DOX—doxorubicin; DCM—dichloromethane; CuS NPs—copper sulfide nanoparticles; CAM—chloramphenicol; DMSO—dimethyl sulfoxide; PLLA—poly(L-lactide); SLES—sodium lauryl ether sulfate; SF—silk fibroin; HFIP—hexafluoroisopropanol; PVP—polyvinylpyrrolidone; PAN—polyacrylonitrile; DXM—dexamethasone; PMI—perylene monoanhydride; EC—ethyl cellulose; KET—ketoprofen; PEO—poly(ethylene oxide); GAA—glacial acetic acid; Ac—acetone; PLGA—poly(d,l-lactic-co-glycolic acid); RhB—Rhodamine B; FE—computational finite element; TFE—2,2,2-trifluoroethanol; mGLT—methacrylated gelatin; GN—gelatin; FITC—fluorescein isothiocynate; HFP—1,1,1,3,3,3 hexafluoro-2-propanol; CA—cellulose acetate; PVP—polyvinylpyrrolidone; KET—ketoprofen.

## 3. Wet-Spinning

### 3.1. Principle and Setup

**Figure 6:**
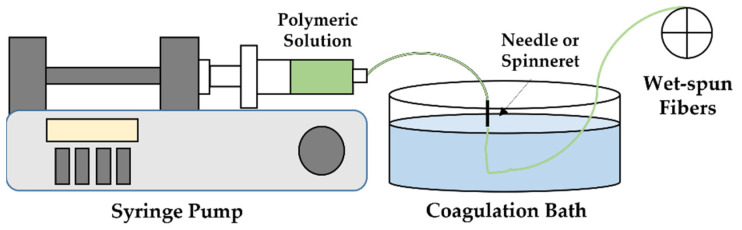
Schematic representation of the wet-spinning apparatus.

Wet-spinning was used for the first time in the textile industry, in the 1930s, to produce synthetic polymeric fibers, namely nylon and spandex [133]. This technique is based on a non-solvent-induced phase inversion principle, in which a polymeric solution is extruded into a coagulation bath composed of a poor solvent or a non-solvent/solvent mixture of the polymer that precipitates in the form of a filament with a diameter of tens to hundreds of micrometers [24,134]. Wet-spinning is divided into three main phases: (1) phase separation, (2) gel separation and (3) liquid crystal spinning. During phase separation, after the polymer fluid is injected into a non-solvent coagulation bath, solvent extraction takes place (miscibility with the bath), resulting in a semi-solid fiber. Afterwards, during gel separation, polymer precipitation occurs, caused by intermolecular bonds, like ionic crosslinking induced by a salt or reacting agent. Finally, during liquid crystal spinning, sufficient alignment and cohesiveness is provided by the formed lyotropic crystalline solution, giving rise to a solid crystalline phase along the wet-spun fibers [135]. The usual wet-spinning setup consists of a syringe mounted on a pump connected to an extruding needle or spinneret that is immersed within a coagulation bath (Figure 6).

Wet-spinning enables the processing of polymers that can neither be melted nor heated at elevated temperatures, overcoming many viscosity issues encountered in other spinning techniques (i.e., melt spinning) [134,136,137]. Wet-spun fibers display high porosity and large diameters (microscale), giving rise to scaffolds with a porous, interconnected architecture that favors cell penetration, adhesion and proliferation [3]. A wide variety of morphologies, sizes and fiber properties can be achieved, depending on the design of the spinneret, composition of coagulation bath, drying temperatures and the polymeric solution components and concentration [24].

Wet-spinning can generate hybrid structures with several levels of organization, dimensions and chemical and physical properties, facilitating cell infiltration and maturation, resembling scaffold micro-environments to physiological conditions [15,138]. With such a technique, difficulties related to polymer thermal degradation, which usually occur while using melt-spinning, are overcome and fibers with diameters from tens to hundreds of micrometers are achieved [139,140]. In the wet-spinning technique, it is possible to produce individual and collectable fibers to be drawn out and subjected to mechanical testing, whereas mechanical testing of individual electrospun fibers is not so feasible. This technique is also considered to be very similar to conventional microsphere-based drug encapsulation methods, avoiding the risks of thermal denaturation of therapeutics. Therefore, a wide range of therapeutic agents, including antibiotics, antimicrobial peptides, proteins, growth factors, genes and viruses have been successfully incorporated onto wet-spun fibers [24]. Still, limitations remain associated with this technique, namely the low production rate, the need for more than one coagulation bath for the complete removal of the solvent from the polymer, and the difficulty in controlling fiber cross-sections due to inward and outward mass transfer, as aside from the elevated costs associated with the process [14]. To date, there have only been few reports on the successful production of wet-spun fibers for biomedical applications, something that can be explained by the complexities related to the number of processing parameters that must be optimized for a consistent and efficient formation of different structures within specific coagulation baths [24]. However, due to the potential and numerous advantages of the constructs originated from the wet-spinning technique, it is predicted that in the next years, research will expand in this field.

For a successful wet-spun fibrous structure to be obtained several parameters must be regulated. The solution properties, including the material concentrations, viscosities, surface charges, surface tensions, polymer natures and functionalities, strongly influence the spinning process. Other features consist of systematic variables, such as the injection rates, take-up velocity, drawing velocity and coagulation bath components. Environmental conditions, including temperature and post-spinning conditions, can also influence the process and, consequently, affect the properties of the fibers. However, the properties of the spinning solution, namely concentration, pH and temperature, are considered the effectors of the final product, not only by determining the fibers properties but also by establishing the efficiency of the fibers production [24,141].

Coagulation kinetics also influences the morphology of wet-spun fibers. Usually, slow solvent/non-solvent diffusion leads to uniform porous structures, since the use of a non-solvent with high coagulation power generates dense core-shell structures, originated from fast surface coagulation and solvent and non-solvent entrapment within the precipitating filament. Additionally, parameters such as the solvent/non-solvent system, temperature, polymeric concentration and molecular weight and the presence of additives in the solution and coagulation bath, nozzle diameter and injection rate, can also significantly affect the kinetics of coagulation [142].

### 3.2. Fiber Structural Organization

**Figure 7:**
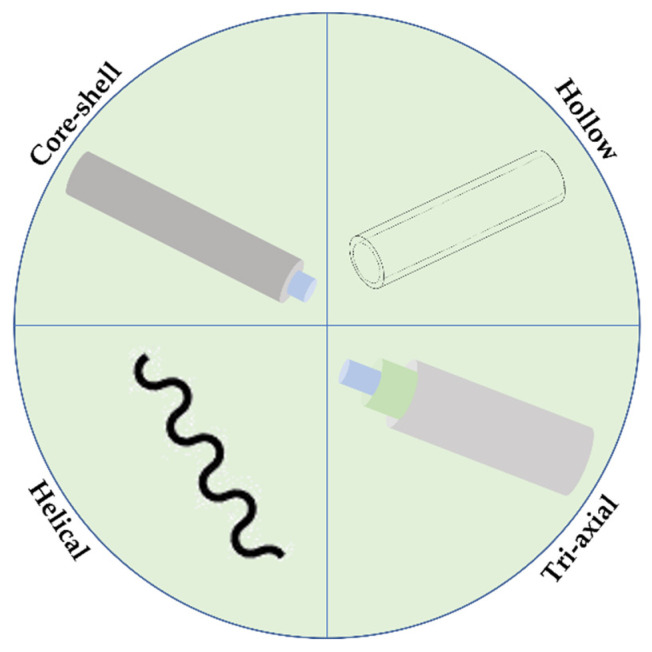
Schematic representation of the most common wet-spun fibers structural organizations.

**Figure 8:**
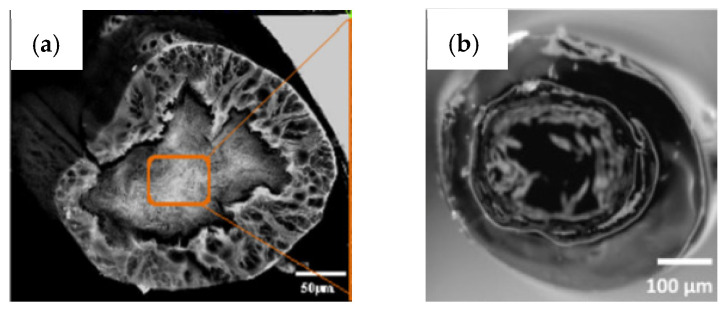
SEM micrographs of (**a**) core-shell and (**b**) tri-axial wet-spun fibers (adapted with permission from [145], published by Wiley, 2015 and adapted from [146], published by MDPI, 2020).

Aside from the conventional wet-spinning approach that generates uniaxial and helical fibers (continuous fibers with a curvy structure), co-axial approaches can also be employed, producing fibers with more complex organizations (Figure 7). In the co-axial method, two polymeric solutions are injected together through a co-axial spinneret and co-extruded into a coagulation bath, originating a core-shell structure (Figure 8a). In case a hollow interior is intended, co-axial wet-spinning approach can also be applied, yet, in these situations, the coagulation bath (or air) is injected throughout the inner port of the spinneret and a polymeric solution is injected through the outer port. The setup for this technique includes two injection syringes and pumps, connected to two ports, a coagulation bath, and a stretching collector. More specifically, the solution of the core component is injected through the inner port of the co-axial spinneret and finally extruded through the inner nozzle into a coagulation bath. In parallel with this mechanism, the shell solution is extruded as the shell of the fiber is being injected through the outer port, enabling the extrusion through the outer segment of the spinneret nozzle [24,143,144]. Co-axial wet-spun fibers, in configurations of core-shell or hollow, have been applied, for instance, in the production of yarn supercapacitors for high-energy density and safe wearable electronics, electronic textiles, sensors and actuators, tissue engineering, medicine regenerative and drug delivery systems [144]. Between the two configurations, hollow fibers are reported to serve a wider range of medicine and biochemistry areas, particularly as drug carriers [143]. In addition, a tri-axial wet-spinning method can also be employed, producing tri-axial fibers (Figure 8b). Here, a tri-axial spinneret is used, containing three input ports, enabling the injection of three polymeric solutions or, in the case a hollow interior is intended, two polymeric solutions with a middle/inner layer made by the passage of the coagulation bath (or air) [24].

#### 3.2.1. Helical

**Figure 9:**
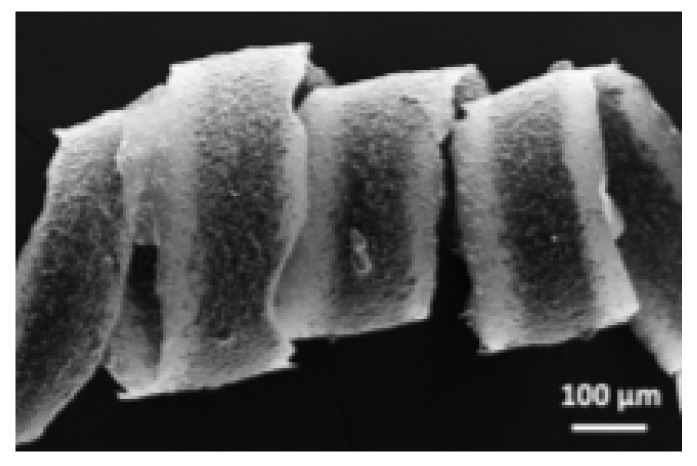
SEM micrographs of helical wet-spun fibers (adapted from [21], published by Beilstein-Institut Zur Forderung der Chemischen Wissenschaften, 2020).

Helical structures (Figure 9) can be found in a wide variety of length scales, varying from the molecular level to the macroscale. Helical shapes can contribute to motion driven processes and structural reinforcement, because of their ability to store mechanical energy and to optimize the surface area. Hence, helical structures are becoming highly attractive for biotechnological and tissue engineering applications and have been used as actuators for microswimmers during gene, drug and chemical delivery, as scaffolds for microtissue constructs, and as a relevant tools for understanding the fundamentals of biological motion and structure formation [147]. The use of wet-spinning technique to produce helical fibers for biomedical applications has only started in recent years. However, it is expected that, in the future, studies with wet-spun helical fibers for tissue engineering or drug delivery will increase significantly.

Wet-spun helical chitosan microfibers loaded with magnetic nanoparticles and coagulated in an ethanolic bath have been researched as actuator systems. Results demonstrated the elevated potential of the engineered fibers for magnetic tissue engineering applications, namely as magnetic and motion-activated cell scaffolds [21]. The number of magnetic particles within the fibers was reported to influence their magnetic properties, being fundamental for reeling the fibers into a helical structure. However, disordered fiber mats can be obtained after collection from the coagulation bath. During the elastic regime, the mechanical properties of the fibers are not dependable on the presence of magnetic particles. However, in the plastic regime, their presence may stimulate earlier fractures. Stretching and chemical crosslinking also influences the Young’s modulus of the fibers. In fact, those parameters, along with post-drying, can tune the mechanical properties of the helical fibers, achieving mechanical features resembling tissue environments [21].

#### 3.2.2. Core-Shell

Core-shell fibers are composed of two separate fiber layers: (1) the outer layer, usually named as “shell”, and (2) the inner layer, also known as “core”, which is completely encircled by the outer layer. Co-axial wet-spinning method is often applied to develop this type of fiber construct [24,144,148].

In biomedicine, core-shell fibers are frequently designed for two main purposes, controlled release of drugs and biomolecules, and/or for local delivery of drugs, being linked to tissue engineering and medicine regenerative applications. Core-shell fibers can preserve the bioactivity of incorporated biomolecules and control their release to targeted microenvironments, to achieve efficient therapeutic effects. Drug initial burst release can be controlled by the incorporation of bioactive molecules at the core, whereas the shell works as a barrier to control and dictate the release of the incorporated biomolecules. As a result, the shell material selection is crucial to lead to desired release profiles and to allow for a proper adhesion of the core-shell fibers [24,148].

Bicomponent fibrous systems, containing two derivatives of hyaluronan, have been produced via the co-axial wet-spinning method, in which a water-soluble hyaluronan was employed at the core, carrying an active substance, whereas a different derivative of hyaluronan, insoluble in water, formed the shell, maintaining the mechanical compactness and structure of the fiber under wet conditions [149]. In another study, Tang et al. developed a carbon nanotube composite surrounded by an insulating shell, like conventional electrical cables, with excellent electrical conductivity. The core-shell fibers presented ultra-high stretchability, excellent stability, fast response, and good washability. Results also indicated that the proposed strain sensor had the potential to be applied in health monitoring, human-machine interfaces, soft robotics and wearable electronics [150]. Core-shell hydrogel optical fibers can also be produced by co-axial wet-spinning, with tunable mechanical and optical-propagation properties. The core component was composed of poly(ethylene glycol diacrylate-co-acrylamide), whereas the shell layer contained calcium and alginate. Data demonstrated low optical attenuation, excellent biocompatibility and tissue-like Young’s modulus, revealing great potential for uses in deep-tissue therapy and brain optogenetic applications [151].

Studies on the optimization of processing and solution parameters have been published on core-axial formulations. Ng et al. produced silk fibroin (SF)-based wet-spun core-shell fibers with a polyacrylamide (PAAm) solution at the inner core and SF/PU dopes at the outer shell. The solutions viscoelasticity affected the spinning behavior of the shell polymer dope which, in turn, depended on the interaction between polymer chains via secondary bonds or physical entanglements. A small change in the degree of interaction between the polymers strongly affected the solution viscosity. This indicates that the influence of the shear flow at the spinneret cannot not be underestimated. The core dope displayed non-Newtonian shear-thinning behavior at a temperature of 80 ºC, since the PAAm chain entanglement stimulated the formation of a network structure. By increasing the shear rates, the mechanical force applied in shearing led to the disruption of aggregates, decreasing the solution viscosity. Still, when using higher polymer concentrations, the amount of entanglements increased, which was then reflected in an augment of the viscosity [152]. The importance of matching the viscosities of core and shell solutions is essential, since the interfacial shear stress variation is minimized when matching viscosities; this also prevents flow buckling and fiber deformation [153].

The effect of shell solution concentrations has also been evaluated. At low concentrations, fibers cannot be formed, giving rise to dispersed floccules, or fibers are formed with weak and irregular cross-sections. To the contrary, at high concentrations, the fibers tend to be very irregular in surface, being highly folded. The fast coagulation of the dope can lead to the production of brittle fibers for post-drawing, with reduced stretchability [154]. Defining optimal concentration rates is therefore essential for a proper fiber production. In a study by Mirabedini et al., co-axial wet-spun fibers were produced from a blend of chitosan with calcium chloride at the ore and alginate at the shell. The spinnability of both sodium alginate and chitosan solutions was analyzed. Results indicated that sodium alginate with concentrations below 2% *w*/*v* could not generate continuous fibrous structures; however, by using concentrations between 2 and 4% *w*/*v*, the solution was highly spinnable. Above 4% *w*/*v*, the polymer solution became too viscous, rendering it as unspinnable [24]. A suitable coagulation bath can guarantee proper rates of solvent extraction and shell solidification. For instance, in case the shell solidification is slow, the fiber cross-section becomes irregular. Yet, when shell solidification is fast, the fiber becomes brittle and hard to be drawn for chain orientation and crystallization. It has also been seen that by increasing draw ratios, fiber surfaces can become smoother, displaying uniform surface morphologies, due to uniform molecular chain alignment while suffering post-drawing [152].

#### 3.2.3. Tri-Axial

Tri-axial wet-spun fibers can be engineered by the wet-spinning using a tri-axial spinneret, which contains three input ports. Using this approach, three different polymeric solutions can be injected simultaneously or hollow sections can be created in the inner regions (middle or core layer) by resorting to the injection of pressurized air or coagulation bath [24]. Tri-axial wet-spun fibers have not yet been reported in biomedical applications. However, these structures have already been used in other fields demonstrating their potential for biomedicine.

Mirabedini et al. developed tri-axial fiber nanostructures, comprising an inner layer of poly(3,4-ethylenedioxythiophene) and polystyrene sulfonate (PEDOT/PSS), coated with chitosan shell, which was wrapped within a carbon nanotube-based fiber. The produced supercapacitor for wearable electronics applications presented high flexibility, delivering a high maximum energy density and power, also showing cyclic stability and remarkable capacitance, marking a potential future generation of wearable energy storage devices. Here, the viscosity was established as a crucial parameter for the spinning solutions properties selection. With co-axial or tri-axial wet-spinning, the viscosity of the outer layers is important since it is intended to provide a protective coating towards the innermost layer. It was observed that, by increasing shear rate, the viscosity decreased, indicating a shear-thinning behavior. Furthermore, the addition of sodium chloride can lead to a decrease in the solution viscosity. The presence of a hydrogel-like shell can lead to an increase of Young’s modulus. The modulus of hybrid materials is influenced by the interactions between the existing layers in the material, since the load distribution efficiency is determined by the adhesion between the components. Overall, in order to achieve proper spinning in all layers, viscosities must be compatible, meaning similar between layer components [146]. In another study different testing concentrations were examined to determine the ideal for the core portion of a tri-axial fiber, in which chitosan was the key element. Concentrations on the range 2–15% *w*/*v* have been reported as spinnable for chitosan [137,155]. The concentration of 3% *w*/*v* has been established as optimal for gel formation with high ionic conductivity when the polymer is processed by wet-spinning, whereas the concentration of 2.5% *w*/*v* has been found ideal as a spinnable dope for the production of continuous fibers. Still, fibers made by chitosan present a non-integrated texture. Thus, other polymers must integrate the mixture to generate a sustainable, resilient core. PEG is one of the best options, since it does not affect the polymer blend spinnability and can generate uniform core textures enclosed within different outer shell components [156].

The production of tri-axial fibers by wet-spinning is very recent, consequently there are very few studies reporting on this new type of fiber architecture. However, the potential of these fibers is clear. This structure allows the incorporation of various materials and biomolecules simultaneously, creating fibers that can deliver/present several biological activities via an all-in-one, multistep formulation.

#### 3.2.4. Hollow

Hollow fibers present a hollow interior, surrounded by a polymeric shell. The procedure for achieving a hollow structure is similar to the co-axial wet-spinning method: the shell spinning solution is fed by a chamber to the external spinneret nozzle through injection, while the core fluid is replaced with pressurized water or the coagulant bath and injected through the inner nozzle [143]. While the spinning process occurs, the take-up velocity is kept with a similar value to the extrusion speed of the fiber, to prevent stretching of the fiber. That way, no external elongational stresses are applied to the fibers, apart from gravity. In addition, the wet-spun hollow fibers usually suffer solvent exchange and other post-treatment procedures to remove residual solvents, thus preventing shrinkage of the fibers, reducing pore collapse and eliminating possible defects, prior to drying [157].

Research has been developed towards the production of hollow fibers for storage, adsorption, and electrode applications. Jia et al. produced lignin-polyacrylonitrile hollow fibers that present great potential for a wide range of applications, including micro reactors, and channel for gases or liquids transfer [143]. Polyacrylonitrile hollow fibers have also been developed for storage and adsorption devices [158] and hollow graphene fibers have been proposed for flexible wearable applications [159]. In recent years, research on the use of hollow, wet-spun fibers for biomedical applications has arisen and it is predicted that. in the following years, much research will evolve towards that direction. For instance, Polacco et al. engineered a hollow PLGA fiber for a controlled drug release system [160], while Lee et al. proposed hollow wet-spun fibers of chitosan-alginate as a new method for encapsulating human hepatocellular carcinoma cells [161].

Studies on the influence and optimization of technique parameters on the properties of the hollow fibers have been published. Wang et al. demonstrated that, by increasing the concentration of the PAN solution at the shell, the porosity of the hollow fiber also enhanced. Pores on the fiber wall form during coagulation, as non-solvent diffuses within the spinning dope. As soon as the stock solution drops into the coagulation bath, both layers (shell and material required for giving rise to the hollow core) undergo double diffusion; the PVP solution at the core dissolves, while the coagulation bath infiltrates the hollow cavity generating pores from within the shell. In addition, capillary condensation and evaporation occurred under different pressures, indicating that the fiber wall presents a mesoporous structure, in which the pore structures are connected to each other, forming a network. Changes in concentrations and ratios between core and shell solutions, feeding speeds and the coagulation bath concentration did not compromise the polymers crystal structure. Yet, by increasing the concentration of the outer solution, the arrangement of the fiber macromolecules becomes denser, stimulating molecular chain crystallization and forming more crystalline areas and, hence, the Young’s modulus [158]. In other research aiming at developing lignin/PAN hollow fibers, decreasing the solution viscosity was seen to decrease the polymers storage modulus and loss modulus (formation of a gel structure). Additionally, by increasing the shear rate, the viscosity of the spinning dope also decreased. Here, dimethyl sulfoxide (DMSO) or formaldehyde (HCHO) were incorporated within the coagulation solvent without affecting the diameters of the fibers and the wall thickness. Yet, depending on the volume fraction of DMSO in the coagulation bath, the porosity and number of surface defects detected on the fibers altered. For instance, when DMSO content augmented, polymer coagulation slowed down, resulting in less porous structures and fewer surface defects. In addition, the strength of the resultant fibers was improved [143].

### 3.3. Tissue Engineering and Drug Delivery Applications

Wet-spun fibers have been gaining considerable attention towards scaffolding substrates over the past years. These fibers enable the attachment, migration, alignment, proliferation, and differentiation of cells, which constitute desirable features for tissue regeneration. In fact, wet-spun fibers have been studied for potential uses in vascular and musculoskeletal tissue engineering and for wound healing purposes (Table 2) [133].

Drug delivery systems effectiveness depends significantly on the drug carrier choice, to guarantee that the active biomolecule reaches the target. Using microfibers as drug carriers is gaining more attention every day because of these fibrous systems improved mechanical properties, easy fabrication, and controllable release profiles. Regarding the last, guaranteeing lower initial burst release rates has been one of the most important features of these systems as compared to spherical vesicles. Moreover, the cylindrical shape of the fibers increases the surface area to volume ratio of the system, which allows drug release over broader area. Besides, the length and cross-sectional radius can be tuned according to the solution and processing parameters applied, contrary to spherical vesicles which tend to be more limited [162,163]. There are still not many reports on the production of more complex fiber architects for biomedical applications, due to the complexity of the structures and the number of parameters that have to be optimized. Nevertheless, it is expected that, in the next years, more studies will arise on the production of core-shell and/or tri-axial wet-spun fibers.

**Table 2:** Main applications of wet-spun fibers with various structural organizations and respective solution/processing parameters.

	Active Agents						
Polymers	Name	Characteristics	Structural Organization	Solution and Processing Parameters	Major Findings	Envisaged Applications	Ref.
PLA; PLGA; Alg	Mouse myoblasts	Cells that originate mouse muscle cells	Monolayer (uniaxial)	Alg (2/4% *w*/*v*) was dissolved in deionized water; cells were suspended in HEPES (20/40/60 million cells/mL) and mixed with Alg solution; the solution was extruded at 0.03 mL/min within a 2% *w*/*v* CaCl_2_ coagulation bath; 20% *w*/*v* 75:25 PLA:PLGA solution dissolved in CHF was injected at 0.0301 mL/min in a coagulation bath of isopropanol; fibers were then seeded with myoblasts; a 0.31 mm diameter needle was used.	Improved in vitro proliferation; exceptional migration of cells; superior engraftment of donor cells	Regenerative skeletal muscle tissue constructs	[164]
CA; PCL	Cinnamon leaf oil, Clove oil and Cajeput oil	Essential oils derived from steam distillation of plant leaves	Monolayer (uniaxial)	CA (10% *w*/*v*) and PCL (14% *w*/*v*) solutions were dissolved in acetic acid, separately; the solutions were injected at 0.00835 mL/min into a coagulation bath of ethanol.	Essential oil-loaded fibers eliminated bacteria more quickly than conventional antibiotics, proving their effective potential to replace antibiotics	Drug delivery systems (i.e., essential oils)	[134]
SA/FK	IDM	Non-steroid anti-inflammatory used to relieve pain, swelling and joint stiffness caused by arthritis	Monolayer (uniaxial)	FK (0.4/0.5/0.67% *w*/*v*) was dissolved in 0.5% *w*/*v* NaOH; IDM (1% *w*/*v*) and SA (2% *w*/*v*) were added to the solution; a 3% *w*/*v* CaCl_2_ coagulation bath was used.	IDM release profile increases over time, relieving the gastrointestinal system from side effects	Drug delivery system to relieve the gastrointestinal side reaction of indomethacin	[165]
SA; GN	Nisin Z	Antimicrobial peptide, originated by the substitution of asparagine for histidine from Nisin A	Monolayer (uniaxial)	SA 2% *w*/*v* was dissolved in deionized water, with posterior addition of a GN 1% *w*/*v* solution, previously dissolved in water, in a ratio of 70:30, respectively; A 1.024 mm diameter needle was used, maintaining a collector distance of 3 cm, with a coagulation bath of 2% *w*/*v* CaCl_2_ solution; the spinning solution was injected at 0.1 mL/min; Fibers were then immersed in Nisin Z.	The incorporation of the peptide improved the fibers structural integrity and provided antibacterial effects against *S. aureus*	Tissue engineering	[163]
CHI	IONPs	IONPs display superparamagnetic properties, usually being presented as magnetite or in its oxidized maghemite form	Helical	IONPs (10% *w*/*v*) were suspended in 1% *w*/*v* acetic acid; CHI (30% *w*/*v*) was used as additive; the solution was injected at 0.334 mL/min into a coagulation bath of absolute ethanol; a 0.25 mm diameter needle was employed.	IONPs were distributed in the fiber matrix as large clusters; dried CHI helices presented spring-like elastic behavior; fibers had strong ferromagnetic properties and exhibited a Young’s modulus in the range of wet-spun CHI fibers	Magnetic and motion-activated cell scaffolds	[21]
CHI-PSS; CHI-PAA/PVS	-	-	Core-shell	Core: CHI (1.5/1.0% *w*/*v*) was dissolved in 1% *w/v* acetic acid; the solution was pumped at 1.0 mL/min and 0.5 mL/min, respectively; Shell: PSS (10% *w*/*v*)/PVS (30% *w*/*v*) were dissolved in deionized water; solutions were injected at 0.4 mL/min and 0.5 mL/min, respectively; A coagulation bath of 50/50 *v*/*v* water/ethanol was used and the distance between the nozzle and the coagulation bath was kept at 3 cm.	Fibers mechanical properties were improved by doping PSS with PEO; fibers presented excellent elongation at break	Tissue engineering scaffolds	[166]
PSU	-	-	Core-shell	Core: egg albumen was separated from the eggs and extruded at 0.367 mL/min; Shell: PSU (18% *w*/*v*) was dissolved in DMF and extruded at 0.585 mL/min; A distilled water coagulation bath was used; the inner diameter of the needle was 0.7 mm with a gap between both layers of 0.25 mm.	A dense structure was obtained in the hollow space of the PSU fiber; the albumen fiber presented good gloss and mechanical properties	Tissue engineering scaffolds	[167]
CHI; Alg	-	-	Core-shell	Core: CHI (0.5/1.0/2.0% *w*/*v*) with different amounts of 2% *w*/*v* CaCl_2_; the solution was injected at 0.234 mL/min; Shell: SA (<2% *w*/*v*) was prepared in water; the solution was extruded at 0.418 mL/min; A coagulation bath of 2% *w*/*v* CaCl_2_ was used.	The incorporation of CaCl_2_ at the fiber’s core enhanced the mechanical properties by 260%; cylinder-shaped monofilaments of chitosan coated with alginate were successfully observed	Drug delivery systems	[135]
HA; SH	IONPs; octenidine dihydrochloride	Octenidine dihydrochloride is a cationic surfactant, active against bacteria	Core-shell	Core: SH was dissolved in water; Shell: HA was prepared with IONPs or octenidine dihydrochloride.	Drug release from the core occurred through cracks; this rupture effect has can be used as a trigger release	Drug carrier	[149]
PLGA; Alg	Dexamethasone; dexamethasone-21-phosphate	Corticosteroid, similar to a natural hormone produced by your adrenal glands; dexamethasone 21-phosphate works as an inducer of apoptosis and inhibitor of the sodium phosphate symporter	Core-shell	Core: PLGA (20% *w*/*v*) and dexamethasone (7% *w*/*v*) were dissolved in DMSO (73% *w*/*v*); Shell: Alg (1% *w*/*v*) was dissolved in water and a 0.1% *w*/*v* dexamethsome-21 phosphate aqueous solution was added to the Alg solution; A 0.31 mm diameter needle was used, along with a 5% *w*/*v* CaCl_2_ coagulation bath.	Alg shell delayed dexamethasone release; the core-shell structure presented two stage releases of dexamethasone and dexamethasone-21-phosphate, with minimum initial burst release	Dual drug delivery system	[168]

Abbreviations: PLA—poly(lactic) acid; PLGA—poly(lactide-co-glycolide); Alg—alginate; CaCl_2_—calcium chloride; CA—cellulose acetate; PCL—polycaprolactone; SA—sodium alginate; FK—feather keratin; IDM—indomethacin; NaOH—sodium hydroxide; GN—gelatin; CHI—chitosan; IONPs—iron oxide nanoparticles; PSS—polystyrene sulfonate; PAA—poly(acrylic acid); PVS—poly(vinyl sulfate); PEO—poly(ethylene oxide); PSU—polysulfone; DMF—N,N-dimethylformamide; HA—hyaluronic acid; SH—sodium hyaluronate; DMSO—dimethylsulfoxide.

## 4. Conclusions

Fibrous constructs produced by spinning techniques represent a promising choice for designing tissue engineering and drug delivery devices. Among the available spinning techniques, electrospinning and wet-spinning can be highlighted, due to simplicity of the methods and the ability to control the morphology, porosity and fiber diameter of the engineered scaffolding systems, by adjusting the processing/solution parameters and making use of simple maintenance settings. Many technique parameters govern the fibers mechanical properties, as well as their morphologies and functionalities. Solution properties, including material concentrations, viscosities and polymer natures, strongly influence the spinning process. Others, such as applied voltage, injection rates and coagulation bath, can also dictate the process and, consequently, affect the properties of the fibers. Nevertheless, several processing and solution parameters are not yet fully understood and need to be optimized, including the effect of the type of collector on different fibers architects. In addition, in the case of core-shell and tri-axial fibrous constructs, as several different polymers are used simultaneously, it is necessary to take into consideration the co-blending effect of such polymer properties, before optimizing the technique parameters. Such techniques enable the production of biomimetic fibrous scaffolds that present features that resemble those of the native extracellular matrix, which can facilitate incorporation and recognition of the scaffolds as part of the biological matter and, thus, lead to a quicker tissue/functions repair. Usually, these constructs are designed with pre-established mechanical strength, structural integrity, and large surface area with an open pore structure to respond to local demands. Furthermore, by loading the constructs with biomolecules, the integration of the antimicrobial biomaterials becomes faster and less painful for the patient. However, big challenges remain, including the inability of implantable fibrous systems to capture simultaneously the mechanical and biological properties of the organs and tissues they intend to treat and replace. Tuning their properties via adjusting processing, solution, or environmental parameters, using a combination of natural and synthetic polymers with biodegradable characteristics, is considered the most reliable option. Complex architectures and configurations such as those highlighted in this review can generate systems that respond more intimately to local demands, while guaranteeing the safety of the user [13,118,122,163,168]. Indeed, by introducing drug delivery systems in which drugs are protected by an additional layer can prevent initial burst release and toxic side effects to the human body. Considering that all these configurations, core-shell, hollow, tri-axial, helical, etc., can be achieved by designing new spinnerets and adjusting the collecting systems, one cannot imagine the many innovations still to come on this field but hope they will continue to offer researchers and medical practitioners with more reliable, precise, and multi-functional medical devices.

## Data Availability

Not applicable.
